# Effect of Microencapsulation on Survival at Simulated Gastrointestinal Conditions and Heat Treatment of a Non Probiotic Strain, *Lactiplantibacillus plantarum* 48M, and the Probiotic Strain *Limosilactobacillus reuteri* DSM 17938

**DOI:** 10.3390/foods10020217

**Published:** 2021-01-21

**Authors:** Clorinda Malmo, Irene Giordano, Gianluigi Mauriello

**Affiliations:** Department of Agricultural Sciences, University of Naples Federico II, Via Università 100, 80055 Naples, Italy; clomal@yahoo.it (C.M.); irene.giordano@unina.it (I.G.)

**Keywords:** emulsion, alginate, reuterin, stress resistance

## Abstract

Cells of the probiotic strain *Limosilactobacillus reuteri* DSM 17938 and of the non-probiotic strain *Lactiplantibacillus plantarum* 48M were microencapsulated in alginate matrix by emulsion technique. Survival of microorganisms in the microcapsules was tested against gastrointestinal (GI) simulated conditions and heat stress. Results demonstrated that the microencapsulation process improved vitality of *Lactiplantibacillus plantarum* 48M cells after GI conditions exposure, allowing survival similarly to the probiotic *Limosilactobacillus reuteri* DSM 17938. Moreover, microencapsulation was able to protect neither *Limosilactobacillus reuteri* DSM 17938 nor *Lactiplantibacillus plantarum* 48M cells when exposed to heat treatments. Microencapsulated *Limosilactobacillus reuteri* DSM 17938 cells were still able to produce reuterin, an antimicrobial agent, as well as free cells.

## 1. Introduction

Nowadays, a new approach to the meaning of food is well accepted by society. In fact, food was recently established as a way to improve health [1,2,3], leading to the introduction of the concept of functional foods. Functional foods beneficially affect one or more target functions in the body in a way that is relevant to the state of wellbeing and health or the reduction of the risk of a disease [4]. Today, functional food ingredients include probiotics, prebiotics, vitamins and minerals, which are used in dairy products, sports drinks and baby foods [4,5,6].

The latest revision of probiotic definition was by the International Scientific Association for Probiotics and Prebiotics [7], an update given on 2001 by FAO/WHO [8]. Most probiotics belong to the genera *Lactobacillus* and *Bifidobacterium*, and they are required to be able to survive through the upper GI tract and to colonise the gut. As a matter of fact, the genus *Lactobacillus* has been recently revised and new genera have been proposed [9]. Probiotics possess three mechanisms of promoting human health: (i) providing end-products of anaerobic fermentation of carbohydrates such as organic acids that can be absorbed by the host, influencing human mood, energy level and even cognitive abilities; (ii) successfully competing with pathogens; (iii) stimulating host immune responses by producing specific polysaccharides [10,11]. A low number of living probiotic cells reach the site of action, as demonstrated by high bacterial mortality during the manufacturing process of functional foods and during the passage through the GI tract. Microencapsulation has been proposed as a way to improve cell survival during probiotics incorporation in foods and after ingestion. Accordingly, a recent paper carefully reviewed the microencapsulation of probiotics for food application [12]. Microencapsulation is defined as the technology for packaging solids, liquid or gaseous materials (the so called “core”) in an inert shell, in capsules that can release their contents at controlled rates under specific conditions [13]. Microencapsulation techniques used to protect probiotic microorganisms include spray drying, spray cooling, fluidised bed coating/air suspension, extrusion, emulsion and coacervation [14,15]. In the emulsion technique, the cell-polymer suspension is added to vegetable oil and the mixture is homogenised to form a water-in-oil emulsion. In some cases, emulsifiers are added to form a better emulsion to lower the surface tension, resulting in smaller spheres. Then, the water-soluble polymer must be insolubilised to start the gelification within the oily phase, and the beads are harvested by sedimentation. The size of the beads is controlled by the speed of agitation and can vary between 25 μm and 2 mm [16]. This technique is easy to scale up, and the costs depend on the type of vegetable oil used [17]. Moreover, by using this technique, microorganisms are not stressed, as happens for example in the microencapsulation by spray drying. Several encapsulation agents have been proposed for the oral delivery of live probiotic bacteria [12]. Among these, alginate has been widely used to microencapsulate these live microorganisms, because it is non-toxic, food-grade, bioavailable, biocompatible and cheap. Alginic acid is a polyuronic acid composed of various proportions of 1-4 linked β-d-mannuronic and α-L-guluronic acids. Upon addition of sodium alginate solution to a calcium solution, interfacial polymerisation is instantaneous, with precipitation of calcium alginate followed by a more gradual gelation of the interior as calcium ions permeate through the alginate system [12].

*Limosilactobacillus reuteri* is an obligately heterofermentative species resident in the human GI tract [9]. Some strains belonging to *L. reuteri* species have been used as probiotics for over two decades in a variety of functional foods and healthcare products [18]. The probiotic activity of *L. reuteri* species, both in vivo and in vitro, is partly attributed to their ability to produce 3-hydroxypropionaldehyde (3-HPA) [19]. 3-HPA, also called reuterin, is a broad-spectrum antimicrobial agent produced by *L. reuteri* during the glycerol metabolism [20].

*Lactiplantibacillus plantarum* is a facultatively heterofermentative species [9] encountered in a variety of environmental niches, including cheese, meat, fish, and plants. It has a proven ability to survive after gastric transit and to colonise the human gut [21].

The aim of this study was to protect a non-probiotic strain, *L. plantarum* 48M, comparing to a well-known probiotic strain, *L. reuteri* DSM 17938. Microencapsulation in alginate matrix was performed by emulsion technique to improve bacterial survival during functional food production and digestion. Accordingly, obtained microcapsules were characterised, testing the tolerance of microencapsulated microorganisms to heat stress and GI conditions.

## 2. Materials and Methods

### 2.1. Microorganisms and Culture Conditions

*L. reuteri* DSM 17938 and *L. plantarum* 48M were used in microencapsulation experiments. *L. reuteri* DSM 17938 was isolated from Reuterin™ (Noos S.r.l.; BioGaia AB, Stockholm, Sweden) and cultured in MRS Broth (Oxoid) at 37 °C, checked for purity and maintained on MRS Agar (Oxoid).

*L. plantarum* 48M, belonging to microorganisms collection of Department of Agricultural Sciences, Division of Microbiology, University of Naples Federico II, was previously isolated from fruit samples and characterised. It was cultured in MRS Broth at 30 °C.

*Escherichia coli* 32, *Hafnia alvei* 53M and *Pseudomonas fragi* 25P used in reuterin production test, belonging to the cited collection, were previously isolated from fresh meat samples and characterised [22]. They were cultured in Tryptone Soya Broth (TSB, Oxoid) supplemented with 0.5% Yeast Extract Powder (Oxoid) at 37 °C, 30 °C and 20 °C, respectively.

### 2.2. Microorganisms Microencapsulation

Bacteria cells were microencapsulated in alginate using the method firstly described by Sheu and Marshall [23] and modified by Truelstrup Hansen et al. [24]. Briefly, 100 mL of bacterial strain culture in the early stationary phase were harvested by centrifugation at 6500 rpm for 10 min. The cell pellet was washed once in an equal volume of a sterile quarter-strength Ringer solution (Oxoid) (Ringer) and weighted. The obtained wet cell pellet was suspended in 18 g of sterile 1% alginate (Sigma, product n. A2033) solution. The cell suspension was mixed with 100 g olive oil (Sigma, product n. O1514) containing 0.5% Tween 80 (Sigma, product n. P8074) by stirring at 300 rpm for 20 min. Calcium ions, necessary for the alginate gelation, were added in two consecutive steps: first, 60 g olive oil containing 0.5% Tween 80 and 62.5 mM CaCl_2_, stirring at 300 rpm for 20 min, and then 40 mL buffered peptone solution added with 0.05 M CaCl_2_. Microcapsules were harvested by one-night sedimentation at room temperature and washed once in a double volume of a sterile quarter-strength Ringer.

All experiments described above were performed using freshly prepared microcapsules. All microcapsules samples were routinely kept at 4 °C.

### 2.3. Enumeration of Microencapsulated Cells

Bacteria entrapped in microcapsules were released by pouring microcapsules in phosphate buffer saline (PBS) (0.1 M, pH 7.0) for 10 min followed by gentle shaking. Viable cell counts of microencapsulated microorganisms were performed by serially diluting 1 mL of microcapsules, dissolved in PBS, in quarter-strength Ringer and plate-counting on MRS Agar. Colonies were counted after 48 h of incubation at optimal growth temperature in anaerobic conditions.

### 2.4. Calculation of Microencapsulation Efficiency

After one night sedimentation, three phases were separated: upper oily phase, middle water phase, and bottom microcapsules. Microencapsulation efficiency was calculated using the following formula: N_t_/N_0_ × 100, where N_t_ is cell loads expressed in CFU/mL after microencapsulation and N_0_ represents cell loads expressed in CFU/mL in overnight culture.

### 2.5. Simulated GI Conditions

Simulated gastric conditions were performed using phosphate buffer saline (PBS: 137 mM NaCl, 2.7 mM KCl, 8.1 mM Na_2_HPO_4_, 1.76 mM KH_2_PO_4_) at pH 2.5.

Simulated intestinal conditions were performed using MRS Broth supplemented with 0.3% bile (Sigma, product n. B8381) and indicated as MRS/bile.

In detail, 1 mL of free or microencapsulated cells (sample before exposure) was suspended in 9 mL of PBS at pH 2.5 or MRS/bile and incubated at optimal growth temperature for the tested strain. Incubation duration was 3 and 4 h for simulated gastric and intestinal conditions, respectively. Samples were analysed at the start (T0) and end (T3 or T4) of the incubation period. Surviving bacteria were enumerated by serially diluting 1 mL aliquots of the test materials, pour plating on MRS Agar and incubating at optimal growth temperature for the tested strain, for 48 h in anaerobic conditions. Three independent experiments were replicated. Residual population at T0 and T3 (gastric condition) or T4 (intestinal condition) was compared with population before the exposure, and *t*-test was performed on the mean values to ascertain significative difference (see Section 2.9).

### 2.6. Heat Treatments

To assess heat tolerance of microencapsulated *L. reuteri* DSM 17938 and *L. plantarum* 48M cells, bacteria survival was tested against four time-temperature combinations: 60 °C for 3 min, 70 °C for 2 min, 80 °C for 2 min, 80 °C for 5 min. Samples were prepared as follows: free or microencapsulated cells were 10-fold diluted in MRS Broth and sealed in glass capillary tubes (Ø 2 mm). The tubes were then placed in a thermostatic water bath and treated at the conditions cited above. At the end of the period, glass tubes were removed and rapidly cooled under running tap water.

Aliquots were sampled before and after thermal treatments and counted by serially diluting 1 mL aliquots of the test material, pour plating on MRS Agar and incubating at optimal growth temperature for the tested strain for 48 h. Results were expressed in terms of surviving percentage as N_t_/N_0_ × 100, where N_0_ and N_t_ are cell loads expressed in CFU/mL before and after heat exposure, respectively. Three independent experiments were carried out.

### 2.7. Viable Staining of Microencapsulated Bacterial Cells

Fluorescence microscopy test was used to investigate cell membrane damage after microencapsulation procedure or after exposure of samples to GI conditions and heat treatments. Furthermore, the test showed possible structural changes of microcapsules. Cells were dyed by using LIVE/DEAD^®^ *Bac*Light™ Bacterial Viability Kit (Molecular Probes, Eugene, Oregon) according to the procedure previously described [25]. Briefly, a stock solution of the two fluorochromes was prepared with 0.7 µL of SYTO 9 (green dye) and 1 µL of propidium iodide (red dye) in 330 µL of sterile deionized water. Microorganisms cultures and microcapsules exposed to GI conditions or heat treatments were ready for dyeing. Freshly prepared microcapsules, instead, were threefold diluted by adding quarter-strength Ringer. Fluorochromes stock solution (6 µL) was applied to 10 µL of each sample and incubated in the dark for 15 min at room temperature. After staining, samples were observed using a Nikon Eclipse E400 epifluorescence microscope (Nikon, Tokyo, Japan) equipped with an UV lamp and a 40× magnification objective. Images were captured by a Nikon Coolpix 4500 Digital Camera equipped with a microscope adapter.

### 2.8. Reuterin Production Assay

Reuterin production from *L. reuteri* DSM 17938 cells was tested by an antagonistic deferred agar spot test [26]. Ten microlitres of sample (free or microencapsulated cells) were spotted on plates of MRS Agar supplemented with 250 mM glycerol and incubated in anaerobic conditions at 37 °C for 24 h. Then, plates were covered with 10 mL of TSB supplemented with 0.75% agar and 0.5% yeast extract and seeded with 2% of an overnight culture of the indicator strain (*Escherichia coli* 32, *Hafnia alvei* 53M or *Pseudomonas fragi* 25P). After the incubation at optimal growth temperature for 24 h, the antimicrobial activity was observed as an inhibition zone of the indicator organism around the sample.

### 2.9. Data Analysis

Analyses were carried out in triplicate, and cell counts were expressed as geometric means of Log CFU/mL for 3 independent experiments. Statistical analyses were conducted using two-tailed *t*-tests, and significance was declared at *p* ≤ 0.05.

## 3. Results and Discussion

### 3.1. Microencapsulation and Encapsulation Efficiency

Results of microencapsulation experiments for both strains are reported in Table 1. After overnight sedimentation, three different phases were visualised, and an upper oily phase of about 160 mL, a middle water opalescent phase of about 20 mL and finally a bottom jelly water phase of about 30 mL appeared, probably containing the microcapsules. In the upper phase, we were able to recover all the oil used during the microencapsulation process; the solutions of water and CaCl_2_, peptone and alginate were probably distributed in the middle and bottom phase. Then, it could be calculated that during the microencapsulation process, about 10 mL of water probably remained entrapped in the alginate network. The microscopic observation led to the results reported in Table 1. In particular, in the oily phase, neither free cells nor microcapsules were detected; instead, in the water middle phase, only free undamaged cells were visualised. Finally, the bottom phase appeared mainly constituted by microcapsules containing green cells.

The LIVE/DEAD^®^ *Bacterial Viability Kit* (*Bac*Light^TM^) was used to evaluate the microcapsules and cell conditions after the microcapsules preparation. Briefly, the *Bac*Light^TM^ was composed of two fluorochromes: the SYTO 9^TM^ and the propidium iodide. The SYTO 9 can penetrate the cell membrane, dyeing the entire cell green, whereas the propidium iodide can only penetrate the damaged cell membrane and, combined with DNA, dyes the cell red when illuminated with UV rays.

**Figure 1:**
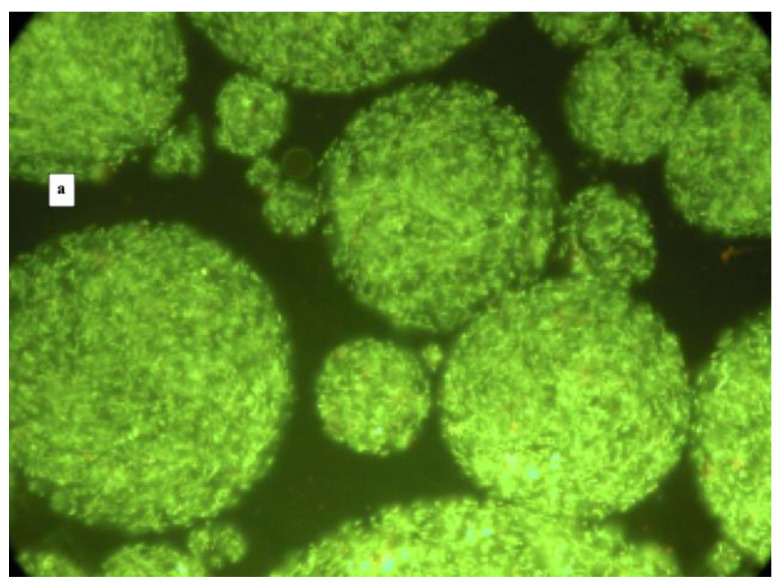

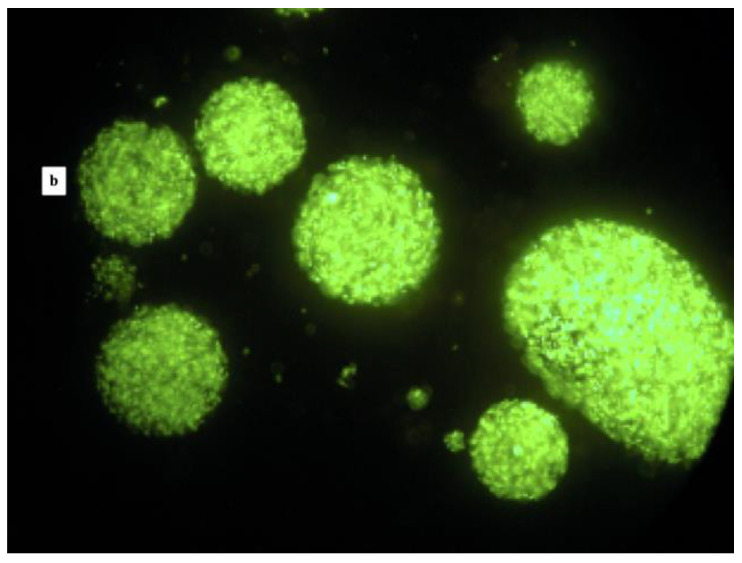
Fluorescence microscopy images at 400× magnification of microencapsulated cells of *L. reuteri* DSM 17938 (panel **a**) and *L. plantarum* 48M (panel **b**) after viable staining.

The observation of microcapsules by fluorescence microscopy showed generally spherical particles; however, some elliptical-shaped capsules were observed as well. The microcapsule size was not homogeneous, ranging from 40 to 150 µm (Figure 1a,b). The cells included in the microcapsules appeared green; therefore, no cell-wall damage occurred during the microcapsule preparation. However, some red cells were also observed, especially for the *L. reuteri* DSM 17938 (Figure 1a), and a few free cells were detected.

Results regarding the shape and size of microcapsules are in agreement with other studies where microcapsules obtained by emulsion technique were analysed. In particular, Truelstrup Hansen et al. [24] reported that microcapsules loaded with bifidobacteria observed by cryo-scanning electron microscopy were spherically shaped with a smooth or rough surface with a visible outline of entrapped bacteria. Similarly, Khalida Sultana et al. [27] obtained spherical and some elliptical beads with randomly distributed bacteria in the alginate matrix. Finally, Valero-Cases and Frutos [28] obtained by emulsion smaller and more irregularly shaped microcapsules containing *L. plantarum* CECT 2020, compared to that obtained by the extrusion method.

Each phase obtained after the microencapsulation process was analysed for viable counting, and results are reported in Table 1. No viable cells were detected in the oily phase, while average values of 7.36 and 4.47 Log CFU/mL were registered in water middle phase for *L. reuteri* DSM 17938 and *L. plantarum* 48M, respectively. Similar differences between the strains were also detected in the counting of microencapsulated cells. In fact, average values of 9.04 and 7.63 Log CFU/mL were registered in the bottom phase for *L. reuteri* DSM 17938 and *L. plantarum* 48M, respectively. Consequently, the encapsulation efficiency for *L. reuteri* DSM 17938 was found to be significantly (*p* < 0.05) higher than *L. plantarum* 48M.

The microencapsulation efficiency was calculated after adjusting the volumes of the phases by applying the formula previously described (Section 2.4). Specifically, the CFU/mL value was adjusted to 100 mL for both the culture and the aqueous phase: 100 mL represents the initial volume of the overnight culture to obtain the microbial pellet used for the microencapsulation procedure. Therefore, the microencapsulation yield was 65.28% for the *L. plantarum* 48M and 91.53% for *L. reuteri* DSM 17938 (Table 1). In the light of the microencapsulation efficiency results, we can hypothesise that different CFU/mL values of obtained phases depend on different compositions of the two strains’ bacterial cell membrane, causing diverse interactions with the microencapsulation agents. As a matter of fact, some authors investigated the interaction between bacterial cells and hydrogels, and they found that even the bacterial growth medium can affect this interaction [29].

### 3.2. Simulated GI Conditions

**Figure 2:**
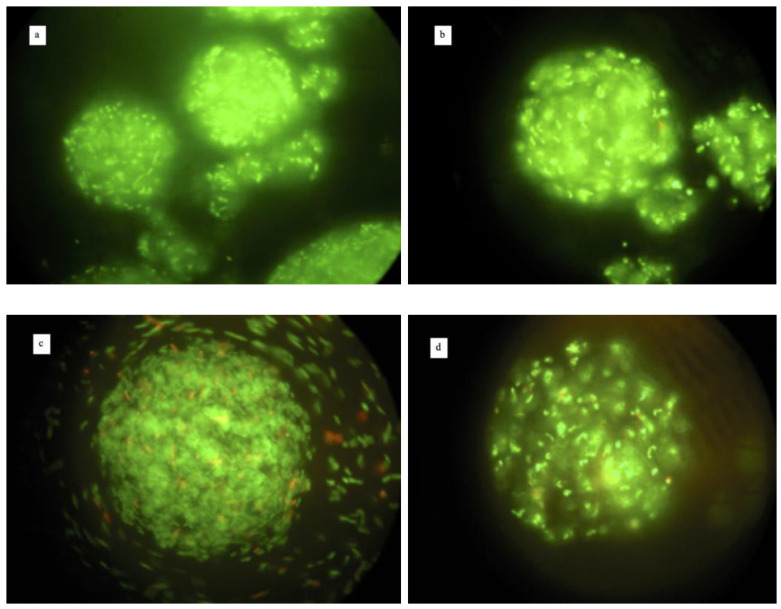
Fluorescence microscopy images at 400× magnification after viable staining of microencapsules of *L. reuteri* DSM 17938 (panels **a**,**c**) and *L. plantarum* 48M (panels **b**,**d**) exposed to gastric (panels **a**,**b**) and intestinal (panels **c**,**d**) conditions.

The microcapsules exposed to the simulated GI conditions were observed by the fluorescence microscopy. The exposure to simulated gastric conditions did not affect the original microcapsules shape; indeed, as Figure 2a,b shows, the cells of both strains were contained in the microcapsules structure and were all dyed green. In contrast, the exposure in simulated intestinal conditions ruined the capsules structure, causing the cells’ discharge. Nevertheless, the cells remained green after 4 h of exposure (Figure 2c,d).

**Figure 3:**
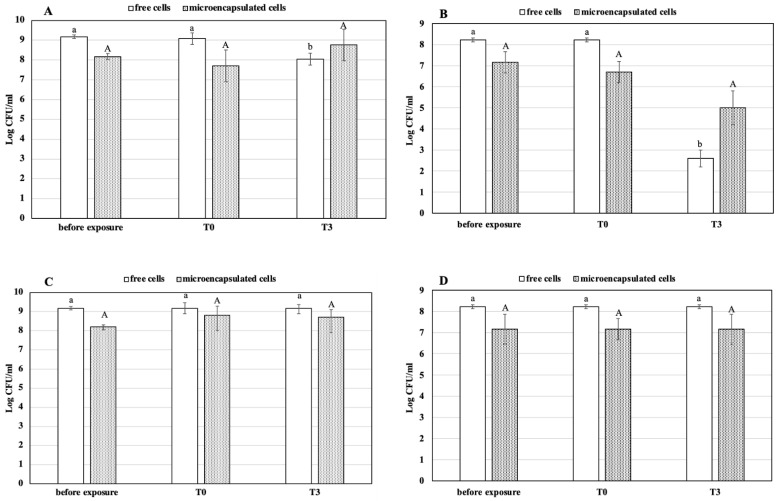
Cell survival of free and microencapsulated cells of *L. reuteri* DSM 17938 (panels **A**,**C**) and *L. plantarum* 48M (panels **B**,**D**) after simulated gastric (panels **A**,**B**) and intestinal (panels **C**,**D**) conditions. Values are means of three independent experiments +/− SD. Different letters (lower for free and capital for encapsulated cells) on the bars indicate significant difference (*p* ≤ 0.05) of the mean values between sample before exposure and samples T0 and T3, as a result of *t*-test.

The results obtained by counting colonies after the exposure to the simulated GI conditions are shown in the Figure 3.

The abilities of the two bacterial strains to survive the gastric condition were markedly different. In fact, as shown in Figure 3A, the free cells of *L. reuteri* DSM 17938 showed about 1 Log reduction after 3 h exposure, compared to the free cells sample of *L. plantarum* 48M, which reduced viability by about 6 Log cycles (Figure 3B). In detail, the population of 9.16 Log CFU/mL before exposure remained stable at T0 (*p* > 0.05), while after 3 h (T3), the mean value was 8.04 (*p* < 0.05). On the other hand, the encapsulated cells did not show any significant reduction (*p* > 0.05) after the treatment. Indeed, microcapsules count was 8.17 and 8.76 Log CFU/mL before and after 3 h of exposure, respectively (Figure 3A). As mentioned above, te Figure 3B shows that the strain *L. plantarum* 48M is very sensitive to a prolonged exposure to the gastric condition. As a matter of fact, the free cell population remained unchanged immediately after exposure (*p* > 0.05), whereas a dramatic reduction by 5.75 Log cycles (*p* < 0.05) of the population occurred after 3 h. On the contrary, the encapsulated cells showed better viability once exposed to the gastric condition. In particular, the Log CFU/mL of untreated capsules was 7.16, and no significant (*p* > 0.05) reduction occurred immediately (T0) and after 3 h (T3) of exposure. Our findings are in agreement with those of other previous works, where microencapsulation in alginate beads was found to increase the survival of probiotics bacteria in gastric conditions [17,30,31,32,33,34,35]. However, to the best of our knowledge, this is the first work in which a non-probiotic microorganism was tested for GI passage resistance in microencapsulated form. Our results clearly demonstrate that there was a slight sensitivity of *L. reuteri* DSM 17938 in a simulated gastric condition; in fact, it is a bacterial probiotic strain already widely used as an oral supplement. This result confirms what we found in our previous work, in which we tested the same microorganism and the microencapsulation by vibration technology to make it even less sensitive [30]. On the contrary, *L. plantarum* 48M showed very high susceptibility to the simulated gastric condition, as demonstrated by strong loss of cell viability. Unquestionably, the microencapsulation protected the *L. plantarum* 48M cells from the detrimental action of gastric juice components and acidity, as demonstrated by the gap of more than 2 Log cycles between T3 samples of free and encapsulated cells. As is well known, resistance to GI conditions is pivotal in the selection of new probiotic microorganisms, making many potential probiotic microorganisms be discarded for their susceptibility to GI transit. Thus, microencapsulation could enhance the number of potential probiotics for food supplementation purposes.

The protective effect also depends on the beads’ size: Lee and Heo [33] reported that the survival of encapsulated cells decreased with decreasing of beads size. Moreover, the concentration of sodium alginate may also affect the survival of cells under acidic conditions, as 1.8% sodium alginate was the optimal concentration among those tested.

The results obtained after exposure to the intestinal conditions did not show any reduction of survival in both strains. An increase in cell load even occurred at the end of incubation period. The number of free cells of *L. reuteri* DSM 17938 showed no significant changes under the same simulated conditions. On the contrary, the encapsulated cells were increased by 0.69 Log CFU/mL at T0 (*p* < 0.05) and remained stable for the whole exposure time (Figure 3C). Similarly, the presence of 0.3% of bile had no lethal effect on the free and encapsulated cells of *L. plantarum* 48M; indeed, the count of the cells in both conditions remained steady after 4 h of exposure (Figure 3D).

The two strains, both free and encapsulated, appeared to be resistant to the intestinal simulated conditions. On the other hand, Muthukumarasamy et al. [36] reported that five strains of *L. reuteri* can survive in presence of 1.2% bile for 6 h. The increased number of CFU/mL of microencapsulated *L. reuteri* DSM 17938 cells under simulated intestinal conditions could be due to the bile salt action. Accordingly, Smidsrod and Skijak-brack [37] demonstrated that the alginate gel is subjected to deterioration when in contact with the monovalent ions, which sequester the calcium ions, causing the cells to be released from the microcapsules. An advantage of this behaviour is that released cells are able to colonise the intestine.

### 3.3. Heat Treatment of Free and Microencapsulated Cells

**Figure 4:**
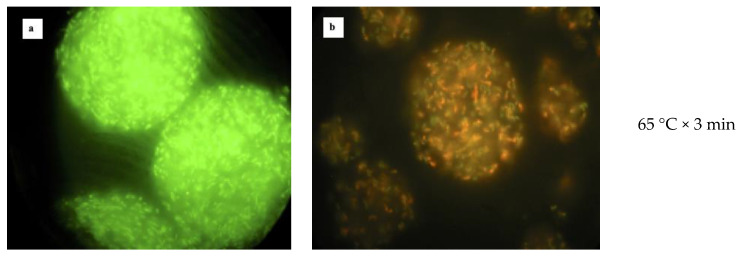

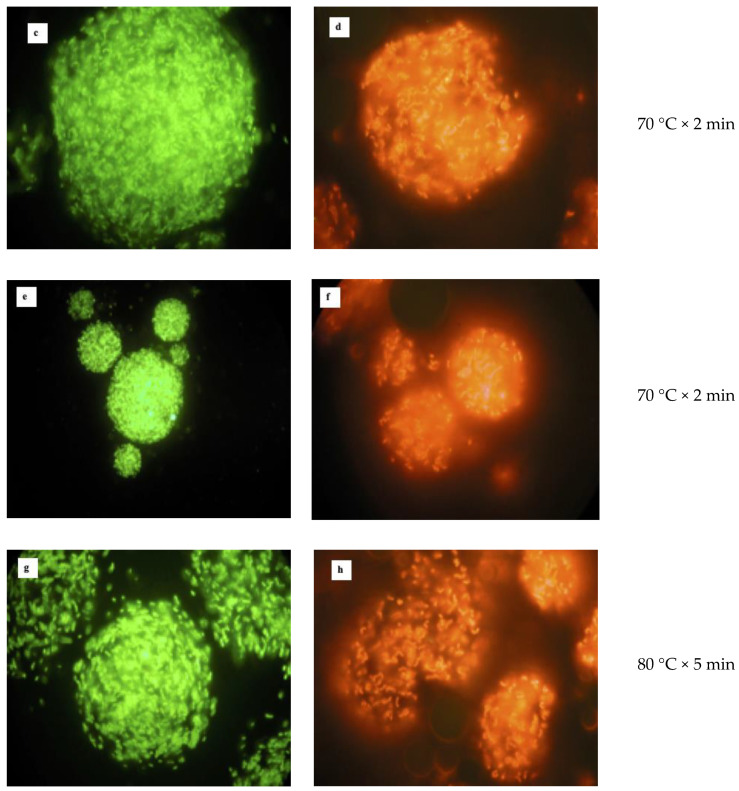
Viable staining of microencapsulated cells of *L. reuteri* DSM 17938 (panels **a**,**c**,**e**,**g**) and *L. plantarum* 48M (**b**,**d**,**f**,**h**) after different heat treatments as indicated on the right sides of panels.

The heat resistance was assessed by exposing free and microencapsulated cells to four time–temperature combinations. The images captured by the fluorescence microscopy show that both bacterial strains had different sensitivity once exposed to high temperature (Figure 4). According to the images, the structure of microcapsules containing *L. reuteri* DSM 17938 (Figure 4a,c,e,g) was not affected by the heat, even though the treatment at 80 °C for 5 min caused the microcapsules damage, with some free cells releasing. At the same time, the cells contained in the microcapsules appeared all dyed green; consequently, no cell membrane damage occurred at any tested temperatures. It is noteworthy that free cells appeared dyed red after treatment at 60 °C for 3 min already (data not shown). Despite the integrity of microencapsulated cells’ membranes, a low survival rate was found after exposing free and microencapsulated cells of *L. reuteri* DSM 17938 to the heat treatments (Table 2). Indeed, at 60 °C, the free cells survival rate was 1.02% and 0.89% for the microencapsulated ones. When the temperature increased by 10 °C, the survival rate was further reduced; in fact, it was 0.0021% for microencapsulated and 0.0042% for the free cells. Finally, no heat resistance was found treating the samples at 80 °C, both for 2 and 5 min; indeed only a few cells survived.

The observation from the fluorescence microscopy also showed no damage to the structure of microcapsules containing *L. plantarum* 48M, and they kept their spherical shape during all treatments (Figure 4b,d,f,h). The main difference from the *L. reuteri* DSM 17938 was the membrane damage after viable staining. Indeed, the cells appeared all dyed red, except for the treatment at 60 °C for 3 min, where some green cells were also detected. Moreover, as shown in Figure 4, as the temperature increased, the red colour became more intensive. This means that the high temperatures damaged the cell membrane of *L. plantarum* 48M. In this case, we could expect a low survival rate considering the colour of the cells after viable staining. In fact, the high temperature dramatically reduced the population load of either free or microencapsulated *L. plantarum* 48M cells. Specifically, the survival rate of *L. plantarum* 48M at 60 °C was 0.0039% for free and 0.0008% for microencapsulated cells. Furthermore, higher temperatures (70 °C and 80 °C) were lethal for the microencapsulated cells, whereas only few free cells survived these treatments (Table 2).

Although it has been well established that the LIVE/DEAD^®^ *Bacterial Viability Kit* (*Bac*Light^TM^) enables differentiation only between bacteria with intact and damaged cytoplasmic membranes, it is often used to differentiate between active and dead cells [38,39]. While it seems accurate to assume that membrane-compromised bacterial cells can be considered dead [40,41], the reverse (intact membranes means active cells) is not necessarily true [42]. Our results seem to confirm these findings; indeed, the cells of *L. plantarum* 48M were found all dyed red, and the results of viable plate count show that there was high mortality after exposure to the heat treatments. Differently, the cells of *L. reuteri* DSM 17938 appeared dyed green even though the plate viable count detected a high mortality. Confirming our results, many authors demonstrated that bacterial cells staining with SYTO9 and Propidium Iodide do not always produce distinct “live” and “dead” populations [40,42,43,44,45,46]. In light of our findings, we can hypothesise a different composition of cytoplasmic membrane of two microorganisms tested, which determined the different behaviour when exposed to the heat treatments.

The results of the present study show that the microencapsulation does not improve the survival of cells once subjected to heat treatments. In particular, the microencapsulated cells of *L. plantarum* 48M were more sensitive than the free ones. After the treatments at 60 °C for 3 min and 70 °C for 2 min, the free cells survived better than the microencapsulated ones (*p* < 0.05). In addition, there was no microencapsulated cell survival (*p* > 0.05) for the treatments at 80 °C for 2 and 5 min. On the other hand, no significant difference was found between the free and microencapsulated cells for all the treatments executed. Accordingly, previous studies show that microencapsulation does not improve bacteria survival after exposure to the heat treatment. Malmo et al. [25] reported that microencapsulation of *L. reuteri* DSM 17938 by spray drying did not improve the heat tolerance when samples were treated at the same time-temperature combinations used in the present study.

The heat tolerance results are dependent on several factors: strain, temperature, microcapsules size, concentration of sodium alginate, etc. In fact, some studies carried out by other authors demonstrated that microencapsulation can improve the cells survival at certain temperatures. Mandal et al. [34] reported that the survival of *Lactobacillus casei* NCDC-298 was improved by the microencapsulation using 4% (*w*/*v*) of sodium alginate; moreover, the temperature was not higher than 65 °C. The improvement of survival was due to the low diffusion of water into the alginate matrix of microcapsules. Similarly, Ding and Shah [47] demonstrated that a lower concentration of sodium alginate (3% *w*/*v*) also improved the survival of lactobacilli treated at 65 °C.

### 3.4. Reuterin Production Assay

**Figure 5:**
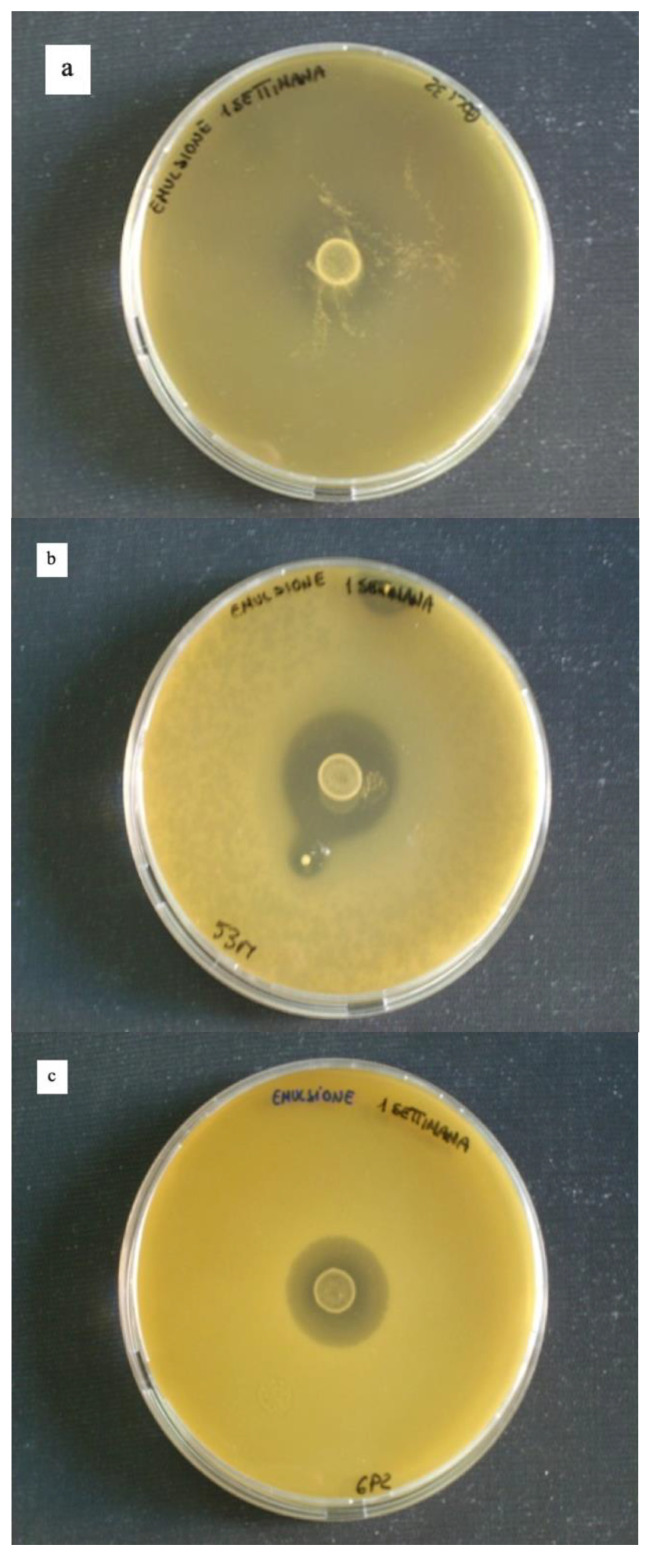
Antimicrobial activity of 1 week old *L. reuteri* DSM 17938 microencapsules against *E. coli* 32 (panel **a**), *H. alvei* 53M (panel **b**) and *P. fragi* 6P2 (panel **c**).

The assessment of reuterin production by microencapsulated cells of *L. reuteri* DSM 17938 was evaluated by performing an antagonist assay using three indicator strains (*Escherichia coli* 32, *Hafnia alvei* 53M and *Pseudomonas fragi* 25P). The microcapsules used to perform the assay were freshly made or kept at 4 °C for 1 week and 4 months. The results showed that *L. reuteri* DSM 17938 kept the ability to produce the antimicrobial agent even after microencapsulation. Moreover, microencapsulated cells produced a clear inhibition zone (Figure 5) also four months after microencapsulation (data not shown). Our results are in agreement with the findings of Malmo et al. [25], who demonstrated that microencapsulated *L. reuteri* DSM 17938 was able to produce reuterin active against *Pseudomonas fragi* 25P.

## 4. Conclusions

The findings of the present study revealed that microencapsulation by emulsion exerts a protective effect on bacterial cells when exposed to GI conditions. In particular, even cells of a non-probiotic strain after microencapsulation by emulsion reached survival levels of a well known probiotic strain. Unfortunately, probiotic and non-probiotic microencapsulated cells did not have a higher percentage of survival when subjected to heat stress compared to free cells.

## Figures and Tables

**Table 1:** Microencapsulation data of *L. reuteri* DSM 17938 and *L. plantarum* 48M. The CFU/mL are adjusted to 100 mL (see Section 2.4).

Phases	Volume of Each Phase mL	*L. reuteri* DSM 17938			*L. plantarum* 48M		
		Fluorescence Microscopy	Log CFU/mL	Encapsulation Efficiency %	Fluorescence Microscopy	Log CFU/mL	Encapsulation Efficiency %
OILY	160	No free cells, no microcapsules	0		No free cells, no microcapsules	0	
WATER	20	Free Cells	7.36		Free Cells	4.47	
CAPSULES	30	Microcapsules with some free cells	9.04	91.53	Microcapsules with some free cells	7.63	65.28

**Table 2:** Survival rate (%) of free and microencapsulated cells of *L. reuteri* DSM17938 and *L. plantarum* 48M after different heat treatments.

Sample	*L. reuteri* DSM 17938				*L. plantarum* 48M			
	60 °C × 3′	70 °C × 2′	80 °C × 2′	80 °C × 5′	60 °C × 3′	70 °C × 2′	80 °C × 2′	80 °C × 5′
free cells	1.02	4.2 × 10^−3^	0.0011	1.9 × 10^−6^	0.0039	3.5 × 10^−4^	7.8 × 10^−5^	3.0 × 10^−5^
microencapsulated cells	0.89	2.1 × 10^−3^	4.2 × 10^−3^	1.3 × 10^−6^	8.0 × 10^−5^	0.0	0.0	0.0

## Data Availability

Not applicable.
